# Viremic non-progression in HIV/SIV infection: A tied game between virus and host

**DOI:** 10.1016/j.xcrm.2024.101921

**Published:** 2025-01-21

**Authors:** Ángel Bayón-Gil, Javier Martinez-Picado, Maria C. Puertas

**Affiliations:** 1 IrsiCaixa Immunopathology Research Institute, Badalona, Spain; 2 Germans Trias i Pujol Research Institute, Badalona, Spain; 3 CIBERINFEC, Institute of Health Carlos III, Madrid, Spain; 4 University of Vic-Central University of Catalonia, Vic, Spain; 5 Catalan Institution for Research and Advanced Studies, Barcelona, Spain

**Keywords:** HIV pathogenesis, viremic non-progressor, VNP, natural SIV host, pediatric HIV infection

## Abstract

High-efficacy antiretroviral treatment (ART) has been a game-changer for HIV/AIDS pandemic, but incomplete CD4^+^ T cell recovery and persistent chronic immune activation still affect HIV-suppressed people. Exceptional cases of HIV infection that naturally exhibit delayed disease progression provide invaluable insights into protective biological mechanisms with potential clinical application. Viremic non-progressors (VNPs) represent an extremely rare population of individuals with HIV, characterized by preservation of the CD4^+^ T cell compartment despite persistent high levels of viral load (>10,000 copies/mL). While only a few studies have investigated the immunovirological characteristics of adult and pediatric VNPs, most of our knowledge about this phenotype stems from its non-human-primate counterpart, the natural simian immunodeficiency virus (SIV) hosts. In this review, we synthesize the insights gained from recent studies of natural SIV hosts and VNPs and evaluate the potential similarities and differences in the mechanisms that underlie the absence of pathogenesis, with special focus on the control of immune activation.

## Graphical abstract

**Figure:**
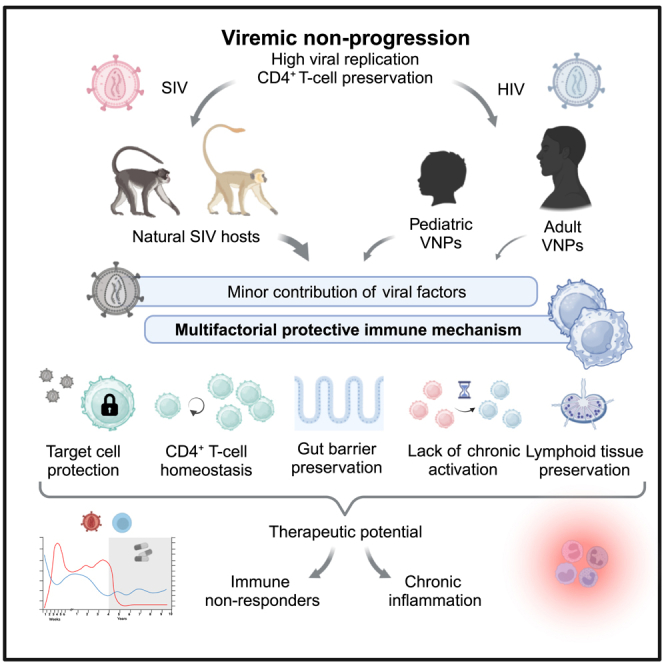

## Teaser

In rare instances, individuals with HIV and certain SIV-infected primates maintain high viral loads without disease progression. This phenomenon, attributed to specific protective immune characteristics, is termed viremic non-progression. Elucidating these mechanisms may inform novel therapeutic strategies to enhance health outcomes in people with HIV.

## Introduction

Acquired immune deficiency syndrome (AIDS) is a disease caused by human immunodeficiency virus (HIV), most predominantly by HIV type-1 (HIV-1). HIV-1 infection is characterized by the progressive loss of CD4^+^ T cells, the main cellular target of infection, and a persistent state of chronic immune activation. The severe and cumulative damage caused to the immune system eventually leads to the emergence of opportunistic infections and cancers that are lethal for the individual. Since the first cases of AIDS were diagnosed in the United States in 1981, the HIV-1 infection has spread globally, becoming one of the most formidable health challenges ever faced by humanity. The development of multiple antiviral drugs that target different steps of the viral replication cycle constituted a turning point in the history of the pandemic. The current antiretroviral treatment (ART), which combines several of these drugs, efficiently suppresses viral replication and stops the deadly progression of the disease. However, despite the invaluable benefit of ART, several problems still persist today. First, a significant proportion of ART-suppressed individuals known as immune non-responders do not recover normal CD4^+^ T cell counts despite control of viremia.^1^ Second, chronic immune activation often persists, which is associated with an increased risk of comorbidities compared to people without HIV.^2^ Third, ART does not cure HIV-1 infection since it does not eradicate the long-lived viral reservoir of latently infected cells.^3^^,^^4^ Therefore, only life-long treatment can prevent viral rebound and the resumption of disease progression. New therapeutic strategies are therefore needed to tackle these yet unsolved adverse events of HIV-1 infection.

A valuable source of inspiration for the development of novel therapeutic strategies is the study of rare cases of people with HIV (PWH) who exhibit atypical disease progression. Probably, the best-known example of these extreme phenotypes of HIV-1 infection with very limited pathogenicity is that of elite controllers. These individuals are able to naturally control HIV-1 replication so they can reduce the plasma viral load in peripheral blood to levels that cannot be detected by routine viral load monitoring (<20–40 copies/mL), and, accordingly, elite controllers show a slower disease progression. Although it is estimated that less than 0.3% of PWH fit the definition criteria of elite controllers,^5^ these individuals have been profoundly studied in order to identify the underlying biological traits that allow them to control viral replication.^6^ Results have shown that a high-quality HIV-1-specific human leukocyte antigen (HLA)-I-mediated cellular immune response by CD8^+^ T cells is likely the main immune factor leading to durable viral suppression. These observations have established robust foundation for research endeavors aimed at replicating the functional cure of HIV-1 infection using various immunological strategies^7^^,^^8^ including therapeutic vaccination, broadly neutralizing antibodies, and immune checkpoint blockers.

**Figure 1:**
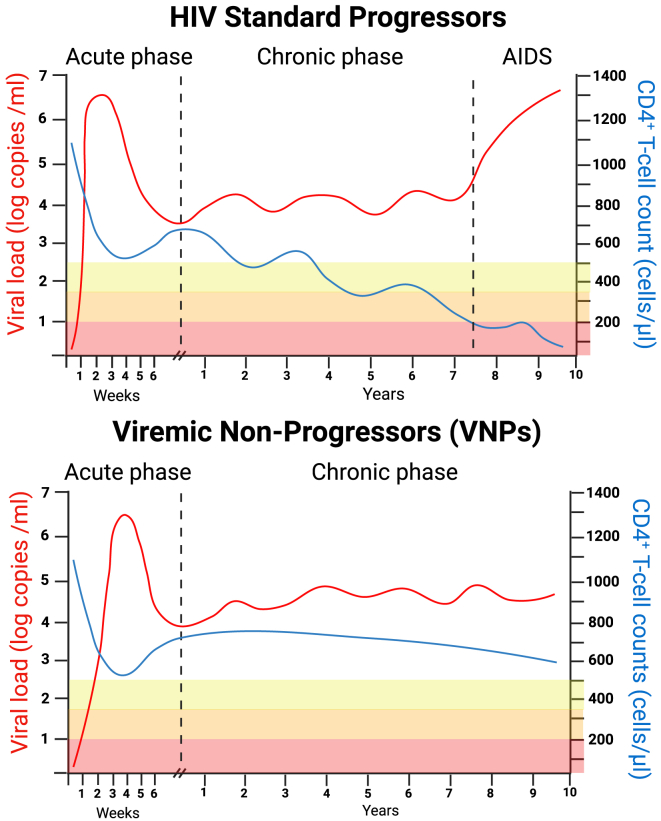
HIV-1 infection profile in standard progressors and viremic non-progressors Chronic HIV-1 infection is characterized by a stable viremia setpoint, which is maintained for years, paralleled by a progressive decline in CD4^+^ T cell counts, which eventually fall below the reference thresholds of 500 cells/μL, 350 cells/μL, and 200 cells/μL. Generally, the magnitude of the viral setpoint during chronic infection is positively associated with the rate of progression to disease, and higher plasma viral loads are associated with faster disease progression. In contrast with the standard progressor profile, VNPs have the surprising capacity to maintain normal CD4^+^ T cell counts (above 500 cells/μL) despite high and persistent levels of viral replication (usually above 10,000 copies/mL).

Interestingly, other extreme phenotypes of HIV-1 infection have demonstrated that absence of pathogenesis can also be achieved despite lack of viremia control, as it is the case of viremic non-progressors (VNPs). These individuals show preservation of normal CD4^+^ T cell counts (>500 cells/μL) despite persistently high levels of viral load (typically more than 10,000 HIV-1 RNA copies/mL) during the chronic phase of the infection (Figure 1). The VNP phenotype is extremely rare in adult populations of PWH (<0.1%),^9^ so only a handful of publications have previously studied the biological characteristics of this interesting phenotype by directly comparing adult VNPs with HIV-1 progressors. Remarkably, the VNP phenotype is more prevalent in children with HIV-1, with an estimated frequency of around 10%, so research on pediatric VNPs has also yielded interesting insights. However, most of our current knowledge on this topic has been derived from studies involving specific non-human primate (NHP) species that can be naturally infected by simian immunodeficiency virus (SIV) and exhibit a similar phenotype to VNPs, characterized by high viral replication and the absence of disease signs. These species are collectively known as natural SIV hosts. Studies on natural SIV hosts, which largely outnumber studies on VNPs, have helped to depict a complex multifactorial mechanism with protective immunological features that mediates the absence of pathogenesis despite persistent viremia. However, a comprehensive understanding of the VNP phenotype, especially regarding human populations, is still missing.

Important considerations should be made for future research on HIV/SIV viremic non-progression. Studies on natural SIV hosts have provided precious insight into the biological intricacies of viremic non-progression, but future research on these models will be restricted given the ethical concerns on the use of vulnerable NHP species for clinical research.^10^ It is also important to highlight that the information we can obtain from animal models is limited, and we should avoid inferring that VNPs perfectly recapitulate the same biological characteristics. Overall, in an effort to shift the focus toward human populations, we advocate revisiting the valuable insights gained from natural SIV hosts and comparing their biological characteristics with those of VNPs. In this review, our objective is to determine the protective factors that are shared between SIV and HIV viremic non-progression, which are likely to hold the greatest therapeutic potential for human populations affected by HIV, and to identify the remaining knowledge gaps related to the immune features of VNPs.

## Infection in natural SIV hosts

There are more than 70 species of NHPs native to the African continent, of which at least 45 are infected by SIV in the wild and are collectively known as natural SIV hosts, including African green monkeys (AGMs) or sooty mangabeys (SMs).^11^ By contrast, no cases of SIV infection have been reported in wild populations of NHPs of Asian or American origin, such as rhesus macaques, despite being susceptible to experimental infection, and, therefore, are known as non-natural SIV hosts. A striking difference between these two groups became noticed soon after their initial description in the 1980s: although both displayed high chronic levels of viral load upon SIV infection, natural SIV hosts lacked the pathogenic CD4^+^ T cell depletion and immunodeficiency observed in non-natural SIV hosts or PWH. A substantial interest emerged in these species, prompting investigations into whether viral or host factors could account for their ability to avert disease progression despite uncontrolled viral replication. Both natural SIV hosts of African origin and non-natural SIV hosts of Asian origin have since been extensively used as models for HIV-1 infection.^12^

### Natural SIV host species

The majority of studies on natural SIV hosts have been performed on AGMs and SMs. AGM is the popular term to refer to primates from the genus *Chlorocebus*. Several species of *Chlorocebus* can be found in extensive areas of the African continent, and captive colonies have been established.^13^ SM is the popular term to refer to NHPs from the *Cercocebus atys* species. Wild SM populations in West Central Africa show a high prevalence of SIV infection, around 50%,^14^^,^^15^ and captive colonies have also been created. Importantly, although extensive research has been historically performed in SM colonies, SM has been listed since 2019 as a vulnerable species by the International Union for Conservation of Nature after documenting an estimated population decline of at least 30% over the last 27 years, which is predicted to continue or accelerate in the following years.^10^ This worrisome situation has led to a moratorium in SM studies involving *de novo* SIV infections, strongly restricting research in this model.

### Progression of the infection

The infection profile of natural and non-natural SIV hosts is different. On the one hand, SIV infection of wild or captive AGM and SM leads to high peak viremia during acute infection and a chronic viral load that remains at high levels,^16^ in the order of 10^3^–10^6^ copies/mL for AGM and 10^4^–10^7^ for SM, which is dependent on the viral strain, the host species, and the availability of target cells.^17^ Importantly, the magnitude of chronic viral load is similar in wild and captive infected animals and approximately 10 times higher than that in average HIV-1 infection.^16^ Despite sustained high viral load, peripheral CD4^+^ T cell counts are minimally impacted and remain relatively stable during chronic infection. Conversely, SIV infection of rhesus macaques with viruses SIVmac251 or SIVmac239 generates robust and reproducible high-level setpoint viremia in the order of 10^5^–10^7^ copies/mL. In this case, uncontrolled viral replication is associated with rapid CD4^+^ T cell count decline, and progression to AIDS within 1–2 years from infection, much faster than average HIV-1 infection.^12^

### Role of virus-related factors in non-pathogenic SIV infection

Multiple studies suggest that SIV infection emerged in natural SIV hosts thousands of years ago, showing an ancient and complex history of virus-host coevolution and cross-species transmission.^18^^,^^19^ By contrast, the emergence of SIV infection in macaque species is recent and was driven by accidental cross-species transmission during laboratory experimentation.^20^^,^^21^^,^^22^ SIVs infecting different NHP species show notable divergence in sequence identity but remarkable similarities in genomic structure. SIVagm, SIVsmm, and SIVmac, which infect, respectively, AGM, SM, and rhesus macaques, together with HIV-1, share a common backbone including the core genes *gag*, *pol*, and *env*; the regulatory genes *tat* and *rev*; and the accessory genes *vif*, *nef*, and *vpr*. Besides this basic structure, SIVsmm and SIVmac genomes encode the accessory protein Vpx, while only HIV-1 encodes the accessory protein Vpu.

The striking difference in the outcome of infection between natural SIV hosts and non-natural SIV hosts could theoretically be explained by attenuation of viruses infecting the former. Multiple studies have also undergone interspecies infections to test viral pathogenic potential. Importantly, most results have shown that macaque species may also lose their CD4^+^ T cells after infection with SIVagm^23^^,^^24^ or SIVsmm,^25^ while natural SIV hosts avoid pathogenesis after infection with SIVmac^26^ or recombinant SIVagm including HIV-1-specific virulence factors.^27^ Collectively, these findings suggest that viruses infecting natural SIV hosts are fully functional and possess pathogenic potential, implying that host factors, rather than viral factors, may play a more important role in the absence of disease.

### Role of host immune factors in non-pathogenic SIV infection

**Figure 2:**
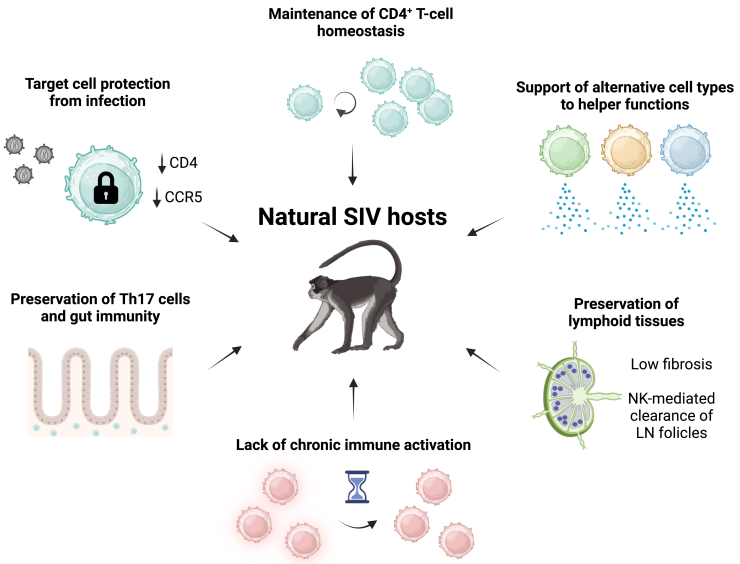
Host immune factors associated with the non-pathogenic phenotype of natural SIV hosts The immune system of natural SIV hosts and their response to infection show characteristics that may contribute to the absence of pathogenesis: target cell protection from infection, support of alternative cell types to helper functions, preservation of gut immunity, lack of chronic immune activation, preservation of lymphoid tissue structure and functionality, maintenance of CD4^+^ T cell homeostasis, and limited role of SIV-specific adaptive immune responses. LN, lymph node.

The immune system of natural SIV hosts and their response to infection show specific characteristics that contribute to the absence of pathogenesis (Figure 2). Protection of target cells from viral infection, maintenance of CD4^+^ T cell homeostasis, and support of alternative cell types to helper functions contribute to minimize the impact of viral infection on immune system functionality. Maintaining the integrity of gut tissue structures and gut immunity serves to reduce the translocation of microbial products into circulation, thereby preventing exacerbated immune response activation and progressive damage to lymphoid tissues. These factors are pivotal in driving disease progression in pathogenic infections. By contrast, SIV-specific adaptive immune responses mediated by cytotoxic CD8^+^ T cells or B cells play a negligible role in controlling viral replication during chronic infection and preventing disease progression, as suggested by experimental depletion studies.^28^^,^^29^^,^^30^^,^^31^^,^^32^

#### Target cell protection from infection

The first step in the viral replication cycle is the entry of the virus into target cells, which is mediated by the interaction of Env proteins with cell surface receptors. Therefore, the binding properties of Env determine the viral tropism, that is, the range of cells that are susceptible to infection. HIV-1 uses CD4 as viral receptor and CCR5 or CXCR4 as viral coreceptors, with CD4^+^ helper T cells being the main target of infection. SIVagm, SIVsmm, and SIVmac also use CD4 as the viral receptor and CCR5 as one of the main coreceptors, while CXCR4-tropic viruses are not as common as in HIV-1 infection.^33^^,^^34^ Importantly, natural SIV hosts have evolved to reduce the expression of the viral receptor CD4 or the viral coreceptor CCR5 and protect their target cells from infection.

In stark contrast to humans, CD4^+^ T cells constitute a minority of total T cells in adult AGMs. Different studies have shown that AGMs naturally reduce their CD4^+^ T cell compartment with aging to reach only ≈10% of total peripheral T cells in adults by conversion of CD4^+^ T cells into CD4^–^CD8αα^+^ T cells.^35^^,^^36^^,^^37^ This process is triggered by cytokines involved in homeostatic proliferation like interleukin (IL)-2, IL-7, or IL-15^36^^,^^37^^,^^38^^,^^39^ and involves epigenetic mechanisms including chromatin remodeling and increased methylation of the *CD4* promoter.^40^^,^^41^ This specific characteristic of AGM has allowed to drastically reduce the pool of target cells susceptible to SIV infection. Importantly, it has been shown that CD4^–^CD8αα^+^ T cells in AGM can still perform classical helper functions upon *in vitro* activation including production of IL-2, upregulation of activation ligands such as CD40L and transcription factors such as FOXP3, expression of cytokines related to T helper cell type (Th)1/Th2/Th17/ follicular helper T (Tfh) profiles, and response to viruses in an major histocompatibility complex-II-restricted manner, and some of those functions have also been observed in *in vivo* immunophenotypic studies.^35^^,^^36^^,^^42^

Comparison between different NHP species has shown that the CD4^+^ T cells of natural SIV hosts, including both AGMs and SMs, express lower levels of CCR5 compared to pathogenic models^43^ both in peripheral blood and gut-associated lymphoid tissue. This fact has been associated with protection from infection as these species needed one-log higher dose of viral inoculum to become infected intrarectally.^44^ Importantly, CCR5^+^ cells are found in especially low proportions in neonates and infant monkeys compared to adult animals.^45^ This fact would explain why vertical infection is such a rare event in natural SIV hosts compared to pathogenic models (7% vs. 25%–75% and 40% prevalence in SM vs. macaques and humans, respectively).^46^^,^^47^ Moreover, *in vitro* stimulation with concanavalin A or αCD3/CD28 antibodies failed to increase CCR5 expression in CD4^+^ T cells from SMs, especially in the Tcm (central memory) compartment, which showed therefore protection from infection even in the presence of activation signals.^48^

#### Maintenance of CD4^+^ T cell homeostasis

CD4^+^ T cell death and progressive decline during chronic infection ultimately lead to immune failure and disease in HIV-1 and pathogenic SIV infection. The immune system tries to compensate the loss of CD4^+^ T cells by increasing the generation of new mature naive cells at the thymus or promoting the proliferation of already-existing cells. However, increased CD4^+^ T cell turnover is also a double-edged sword, as it gives rise to new target cells that fuel the infection.

Unlike pathogenic HIV-1 and SIV infections, different studies have reported reduced levels of CD4^+^ T cell death during chronic infection of natural SIV hosts.^49^ However, the molecular mechanisms underlying this observation had long remained unexplored. Previous research suggested that pyroptosis of quiescent cells undergoing abortive infection is a major driver of CD4^+^ T cell death in pathogenic HIV-1 infection.^50^ More specifically, a recent work has shown that inflammasome formation and pyroptosis are triggered by CARD8-mediated recognition of viral protease in humans and macaques.^51^ Strikingly, researchers also found that loss-of-function mutations in the *CARD8* gene of AGMs and SMs prevented the correct function of the inflammasome,^51^ potentially limiting CD4^+^ T cell death and contributing to the preservation of this compartment in natural SIV hosts.

It has long been described that pathogenic SIV infection in macaques is also associated with increased CD4^+^ T cell turnover,^52^ but not in non-pathogenic infection in natural SIV hosts.^53^^,^^54^^,^^55^ IL-7 is the most important cytokine promoting homeostatic proliferation of T cells. Early studies on SM and macaques have suggested differences in the kinetics of plasma IL-7 levels between both models, with natural SIV hosts showing peak induction within the first 5 weeks of infection followed by partial control and macaques showing a progressive increase along chronic phase.^56^^,^^57^ Future studies should further clarify with more detail the double-edge role of IL-7 in pathogenic and non-pathogenic SIV infection. Also, bone marrow-associated CD4^+^ T cell proliferation during chronic infection has shown to be important for maintenance of stable CD4^+^ T cell counts in SMs,^58^ while lower rates of infection in crucial CD4^+^ T cell subsets with high proliferative potential, such as stem cell memory (Tscm) and Tcm, have been described as a distinctive feature of natural SIV hosts that may also contribute to preserve intact CD4^+^ T cell homeostasis.^59^^,^^60^

#### Support of alternative cell types for helper functions

CD4^+^ T cell count decline during HIV-1 infection and SIV infection in macaques leads to immune system failure and fatal immunodeficiency. By contrast, CD4^+^ T cells may not be essential for immune functionality in natural SIV hosts. Exceptional cases of SIV-infected AGMs and SMs with total CD4^+^ T cell depletion in periphery have been documented.^61^^,^^62^^,^^63^^,^^64^ Interestingly, some studies have linked this depletion to infection with promiscuous viruses that use multiple alternative coreceptors and can potentially target a wider range of cells.^61^^,^^62^^,^^65^ Strikingly, in some of these cases, despite total lack of CD4^+^ T cells, the animals did not show any sign of disease. Later studies involving experimental CD4^+^ T cell depletion^66^ also showed lack of disease signs in these animals, with preserved levels of immune activation and cell proliferation markers, and absence of lymphoid and gut tissue disruption.

The absence of pathogenesis despite loss of the CD4^+^ T cell compartment might indicate the presence of alternative cell types that support CD4^+^ T cells and perform helper functions without being susceptible to infection, as in the aforementioned case of CD4^−^CD8αα^+^ T cells in AGMs. Similarly, it has been suggested that double-negative CD4^–^CD8^−^ T cells, γδ T cells, or natural killer T cells, which preserve their capacity to secrete cytokines upon SIV infection in natural SIV hosts but not in macaques, can contribute to maintain immune homeostasis upon infection in SMs.^35^^,^^67^^,^^68^^,^^69^^,^^70^

#### Preservation of gut immunity and lack of chronic immune activation

Early events of HIV-1 and pathogenic SIV infections include massive depletion of gut-resident CD4^+^ T cells and local production of inflammatory cytokines. In particular, depletion of CD4^+^ T cells with a Th17 profile, which contribute to develop bacterial and fungal responses, compromises gut immunity. These events lead to intestinal barrier disruption and microbial translocation, which in turn exacerbates the inflammatory milieu and sustain chronic immune activation.

SIV infection in natural SIV hosts is characterized by the absence of damage to the gut epithelial barrier,^71^^,^^72^ lack of expansion of the enteric virome,^73^ and no signs of microbial translocation at either acute or chronic infection.^71^^,^^72^^,^^74^ Surprisingly, studies in AGM and SM have shown that preservation of gut functionality occurs despite acute depletion of CD4^+^ T cells in the gut already in the first month of infection,^75^^,^^76^^,^^77^ in contrast with relatively stable CD4^+^ T cell counts in peripheral blood. However, a remarkable difference is that Th17 cells are specifically preserved in natural SIV hosts and help maintain gut immunity and low levels of immune activation.^77^^,^^78^ Th17 differentiation is promoted by IL-21 and inhibited by kynurenine and other tryptophan degradation products generated by the enzyme IDO1. Preservation of a subset of IL-21-producing CD4^+^ T cells in the gut^79^ and inhibition of IDO1 activity associated with a protective *Lactobacillus*-enriched microbiome^80^ probably contribute to the maintenance of the Th17 pool in natural SIV hosts. Moreover, as mentioned earlier, it is plausible that cells other than CD4^+^ T cells play an important role in maintaining the pool of IL-17-producing T cells. Interestingly, a study has shown that IL-17-producing CD8^+^ T cells (Tc17) are lost through the chronic phase of infection in macaques, but they are preserved in SMs.^81^

The link between preservation of gut immunity and maintenance of low levels of immune activation has been highlighted by different interventional studies. Experimentally induced colitis with dextran sulfate sodium or *in vivo* administration of microbial-derived products such as lipopolysaccharide (LPS) in AGM led to increased plasma levels of proinflammatory cytokines, T cell activation, and CD4^+^ T cell decline.^82^^,^^83^ By contrast, reduction of intestinal microbial content by rifaximin treatment or *in vivo* administration of sevelamer, an LPS-sequestering drug, to the pathogenic macaque model of SIV infection reduced the levels of markers for microbial translocation, inflammation, and coagulation as well as levels of T cell activation.^84^^,^^85^

The production of proinflammatory molecules by monocytes is greatly limited in natural SIV hosts and mostly restricted to the acute phase of infection, with normalized levels of most cytokines during chronic phase and comparable to uninfected animals.^19^^,^^86^^,^^87^ Similarly, the levels of activation, proliferation, and apoptosis in T cells are not increased during chronic infection in natural SIV hosts, either in tissues or in peripheral circulation.^49^^,^^72^^,^^88^ Interestingly, preservation of gut tissue functionality and subsequent lower exposure to translocated bacterial products is not the only factor contributing to attenuate inflammation in natural SIV hosts. Genetic polymorphisms in the *Toll-like receptor (TLR)4* gene that are shared by different species are associated with reduced production of proinflammatory cytokines in response to LPS stimulation.^89^ Other factors likely contributing to the quick resolution of the inflammatory response in natural SIV hosts include lack of expansion of proinflammatory monocyte populations, the presence of tissue macrophages with upregulated transcriptional signatures of regenerative wound healing, expansion of regulatory T cells (Tregs), early induction of anti-inflammatory cytokines such as transforming growth factor β1 (TGF-β1) and IL-10, and increased levels of other immunosuppressive mediators such as adenosine.^90^^,^^91^^,^^92^^,^^93^^,^^94^

The interferon (IFN) response is essential to combat viral infections. In particular, plasmacytoid dendritic cells (pDCs) are the main producers of type-I IFNs during viral infections via TLR7 and TLR9 recognition, playing an important role in antiviral defense and immune activation by inducing the expression of a myriad of interferon-stimulated genes (ISGs) in multiple cell types. Diverse studies have demonstrated that pDCs derived from natural SIV hosts are fully functional. These cells migrate to lymphoid tissues upon acute infection, efficiently sense autologous viruses, and facilitate IRF7-mediated IFNα production at levels comparable to macaques, being the main type-I IFN producers *in vivo* across all these species.^95^^,^^96^^,^^97^ However, studies in SMs have shown that pDCs downregulate IFNα production much faster than in macaques and lack ISG expression during the chronic phase of infection.^96^^,^^98^ Indeed, studies comparing the transcriptional profile in peripheral blood mononuclear cells (PBMCs) and lymphoid tissue of AGMs or SMs and macaques after SIV infection have consistently shown that all species mount a strong and efficient IFN response during acute infection,^99^^,^^100^^,^^101^ but while ISG expression remains elevated during chronic infection in macaques, it is quickly resolved in natural SIV hosts. Although the molecular mechanisms that are responsible for this downregulation have not been clearly identified, negative regulators whose expression is increased during late acute infection only in non-pathogenic species such as *SOCS1*, *ADAR*, *IL-10*, or *LAG3* have been proposed as potential mediators.^9^ Importantly, PBMCs isolated from chronically infected AGM and SM upregulated ISG in response to *in vitro* stimulation with IFNα, showing that they are not refractory during this stage.^101^

#### Preservation of lymphoid tissues

Secondary lymphoid organs like lymph nodes are progressively disrupted during pathogenic HIV-1 and SIV infections. An excessive response of Tregs to counterbalance immune activation leads to increased TGF-β1-mediated fibrosis of lymph nodes, which restricts the access of naive T (Tn) cells to essential survival factors like IL-7 and increases apoptosis.^102^^,^^103^^,^^104^^,^^105^ Importantly, natural SIV hosts lack fibrosis of lymph nodes and show preserved tissue architecture and functionality.

B cell follicles within lymph nodes constitute an immunoprivileged sanctuary for uncontrolled viral replication in pathogenic infections due to the high density of target cells, the restricted access of cytotoxic cells, and the environment favoring immune activation and cell-cell contact. Interestingly, natural SIV hosts also show a remarkable capacity to clear viral infection from these sites, with a preponderant role for terminally differentiated CXCR5-expressing natural killer (NK) cells that can migrate into follicles to clear infected cells.^106^^,^^107^^,^^108^^,^^109^ Remarkably, this NK cell phenotype was successfully induced in SIV-infected ART-treated macaques by combined IL-21+IFNα treatment and was associated with viral clearance in lymph nodes and delayed viral rebound after ART interruption.^110^ Interestingly, NK cell-mediated clearance of infected cells in AGMs also extends to mesenteric lymph nodes in the gut, which was associated with preservation of immunoglobulin A (IgA)^+^ memory B cells and total IgA levels, probably contributing to preservation of gut immunity.^111^

## Infection in pediatric VNPs

### HIV-1 infection in children

Pediatric HIV-1 infection differs from adult HIV-1 infection in at least two main aspects: the mode of transmission of the virus and the characteristics of the host immune system at early stages of life.

HIV-1 can be transmitted from infected mothers to children *in utero*, at birth, or by milk breastfeeding.^112^ The transmission rates depend on the route, with 5%–10% for *in utero* infection, 10%–20% for intrapartum infection, and 5%–15% for breastfeeding. Importantly, the state of the maternal immune system is of key importance for vertical transmission and pediatric disease progression,^113^ as mothers with high viral loads have an increased risk to transmit the virus to their offspring and low maternal CD4^+^ T cell counts have been associated with faster disease progression in their children.

The newborn immune system is characterized by a predominant anti-inflammatory and tolerogenic environment biased toward Th2/Th17 responses, a low innate antiviral type-I IFN response, limited Th1 support, abundance of naive lymphocytes, the absence of immunological memory, and normal CD4^+^ T cell counts exceeding 1,500 cells/μL. These factors collectively increase susceptibility to viral infections and make infants more vulnerable to uncontrolled HIV-1 replication.^112^^,^^114^^,^^115^^,^^116^ As children age and their immune system matures, it gradually acquires typical adult characteristics, including a more robust antiviral response, a proinflammatory environment, and the development of classical Th1-specific responses that mediate antiviral immunity.

#### Progression of the infection

Different studies have characterized the infection profile of pediatric HIV-1 infection.^117^^,^^118^ HIV-1-infected newborns show very high and persistent viral loads, which are typically in the range of 10^5^–10^7^ copies/mL and are only partially controlled after a process that normally takes around 5 years, in stark contrast with the few weeks that are needed in adult infection to achieve a relatively stable setpoint of viral load. The fact that functional HIV-1-specific CD8^+^ T cell responses with high magnitude and breadth are infrequent in children of less than 3 years of age probably explains this observation.^119^ Disease progression induced by HIV-1 infection occurs more rapidly in children than in adults, leading to over 50% mortality among untreated HIV-1-infected newborns within 2 years.^120^ However, cases of slow pediatric progressors have been documented.^117^^,^^121^^,^^122^^,^^123^ Most of these individuals maintain a normal-for-age CD4^+^ T cell compartment despite high levels of viral replication. This VNP phenotype accounts for approximately 10% of pediatric infections, being much more abundant than in adulthood. By contrast, only rare cases of viremia control and elite control have been reported in pediatric infection,^124^ with an estimated proportion of elite controllers around 0.02%–0.08%.

### Viral factors in pediatric VNPs

Viral attenuation has been linked to slow disease progression in pediatric HIV-1 infection in some studies. However, the potential contribution of specific viral restriction factors to different pediatric progression profiles remains largely unknown. In vertical HIV-1 infection, viral fitness is influenced by the immune pressure exerted by the maternal immune system on the viral population at the time of transmission. In this context, some studies have suggested that the presence of protective HLA-I alleles in the mother can induce escape mutations with high fitness cost for the virus, resulting in reduced replicative capacity.^125^^,^^126^ This diminished capacity is also observed in viruses from their respective children and is associated with slower disease progression. Conversely, cases of extremely high viral loads have been associated with rapid pediatric progression.^127^^,^^128^ Despite these observations, the frequent occurrence of high-level viremia in slow progressors has raised questions about the relative importance of viral factors compared to host immune factors in pediatric HIV-1 infection.

### Host immune factors in pediatric VNPs

Previous studies on pediatric populations with different degrees of viremia and rates of disease progression have largely focused on the adaptive branch of the immune response and particularly in the CD8^+^ T cell response and the role of the HLA-I haplotype. As we previously mentioned, the maternal HLA-I haplotype can exert an important effect on pediatric disease progression by conditioning the replicative fitness of the transmitted variants. By contrast, the HLA-I haplotype inherited by their children is not associated with the level of viral load nor the rate of disease progression,^126^ likely because adaptive immune responses in young children generally still lack complete functionality and do not efficiently exert immune pressure on the virus. However, those children carrying protective HLA-I alleles who manage to develop HIV-1-specific CD8^+^ T cell responses against Gag epitopes presented by those alleles tend to show a slower progression profile than those who do not develop those responses.^129^ The protective role that HLA-mediated CD8^+^ T cell immunity may play in some cases of pediatric infection is also supported by an exome sequencing study on hundreds of pediatric non-progressors and rapid progressors that identified several HLA-I alleles associated with slow and fast rates of progression.^130^ Moreover, different studies have shown that the presence of Gag-specific T cell responses with high polyfunctionality and IFNγ production capacity is associated with slow pediatric progression, especially in those cases where viral load was relatively controlled to values below 10^4^ copies/mL^131^^,^^132^^,^^133^^,^^134^^,^^135^ Another study associated relative control of viral load and slower disease progression in children with increased frequency of HLA-I alleles carrying the Bw4 epitope, which are associated with killer-cell immunoglobulin-like receptor (KIR)-educated NK cell response, and NK cell populations with strong responsiveness to cytokine stimulation.^136^ Altogether, and given the well-established role that protective CD8^+^ T cell responses have in adult HIV-1^+^ elite controllers, these results suggest that an important proportion of pediatric VNPs who show slow disease progression thanks to protective HLA-I-mediated immune responses may evolve with age toward a controller-like phenotype with lower levels of viral load.

The role of other cell types has also been addressed in previous studies on pediatric VNPs. HIV-1 infected children generate higher titers of HIV-1-specific broadly neutralizing antibodies and with higher breadth than adults,^123^ regardless of the rate of disease progression. Interestingly, higher titers of Gag-specific antibodies and increased anti-inflammatory properties including higher galactosylation in Fc regions have been associated with slow disease progression.^137^ Finally, interesting parallelisms have been established with infection in natural SIV hosts.^123^^,^^138^ Specifically, the hallmark publication by Muenchhoff et al. including 170 pediatric non-progressors with a pooled viral load setpoint above 10,000 copies/mL over the first 10 years of life showed that normal CD4^+^ T cell counts were associated with multiple protective immune characteristics. Plasma quantification of intestinal fatty acid binding protein (iFABP) and soluble CD14 (sCD14) suggested preservation of gut tissue structures and lower levels of microbial translocation in pediatric slow progressors compared to pediatric progressors. Stratification of participants according to their CD4^+^ T cell counts revealed that those individuals with the higher counts exhibited lower levels of activation across CD4^+^ and CD8^+^ T cell subsets, as well as a higher proportion of CD4^+^ T naive cells and lower proportion of CD4^+^ T memory cells. Importantly, T cell activation was positively correlated with sCD14 levels. Finally, they also described lower expression of the viral coreceptor CCR5 in the CD4^+^ Tcm compartment, probably contributing to the lower observed rates of infection in Tscm and Tcm, two subsets with a high proliferative capacity to replenish and maintain the CD4^+^ T cell compartment.

## Infection in adult VNPs

Several studies have described exceptional cases of adult VNPs, who maintain normal CD4^+^ T cell counts and avoid disease progression despite uncontrolled viral replication, typically above 10^4^ copies/mL. Conservative estimates suggest that less than 0.1% of HIV-1-infected individuals meet these criteria,^9^ but only a few dozen cases have been reported in the literature. Importantly, different terminology and definition criteria have historically been employed to identify this intriguing group of individuals.^139^ As a consensus definition, we propose to use the following selection criteria: mean viral load > 10,000 HIV RNA copies/mL (starting six months after date of seroconversion/diagnose) and CD4^+^ T cell decay rates < 10% cells/μl/year for a minimum period of 4 years since diagnosis of HIV-1 infection or the estimated seroconversion date.^140^ Additionally, the limited number of VNPs analyzed in previous studies and the comparison with control populations with varying characteristics have partially contributed to the inconsistent results observed across different works. Overall, previous studies suggest that adult VNPs may share certain features with natural SIV hosts and pediatric VNPs. However, given the aforementioned limitations, many questions remain unanswered, and a comprehensive understanding of this exceptional HIV-1 infection phenotype remains elusive.

### Viral factors in adult VNPs

The persistently high levels of viral replication during chronic infection suggest that viruses infecting VNPs are fully functional. Different studies have confirmed that viruses from VNPs and progressors encode Env proteins with comparable fusogenic activity and Nef proteins with similar functionality.^141^^,^^142^ Furthermore, both types of viruses show similar *in vitro* infectivity, replication capacity, and CD4^+^ T cell depletion potential.^141^^,^^142^^,^^143^^,^^144^ Therefore, it appears that host immune factors also play a crucial role in the absence of pathogenesis in adult VNPs.

### Host immune factors in adult VNPs

A robust and high-quality HIV-1-specific CD8^+^ T cell response is linked to elite control of HIV-1 infection.^6^ In contrast, the role of HIV-1-specific CD8^+^ T cells appears less significant in the absence of pathogenesis in adult VNPs. Different studies have revealed a limited magnitude, breadth, and cytolytic activity of these cells, along with comparable *gag* mutation rates and intrapatient viral sequence diversity when compared to progressors.^145^^,^^146^ These findings collectively suggest a similar evolutionary pressure imposed on the virus.

The preservation of the CD4^+^ T cell compartment in adult VNPs may be attributed to a more efficient generation of new CD4^+^ T cells in the thymus or the homeostatic proliferation of memory CD4^+^ T cells. While some studies have reported similar proportion of CD4^+^ Tn cells and comparable levels of thymic output between VNPs and progressors,^147^ others have associated this phenotype with preserved generation of Tn cells in the thymus.^148^^,^^149^ Similarly to natural SIV hosts and pediatric VNPs, some studies have also indicated lower infection rates in Tscm and Tcm cells in adult VNPs compared to progressors,^150^ as well as lower expression of CCR5 and higher prevalence of *CCR5Δ32* deletion in heterozygosity.^140^ Interestingly, a recent publication detailing two exceptional cases of VNPs who lost their status nearly after two decades of infection found that the sudden increase in plasma viral loads and drastic CD4^+^ T cell decline coincided with a shift to X4-tropism and increased replication capacity in viral isolates.^151^ This change may have been triggered by the particularly low expression of viral coreceptors CCR5 and CXCR6 observed in the CD4^+^ T cells of these individuals.

The potential role of attenuated chronic immune activation in the VNP phenotype remains a topic of debate. Several studies have shown that VNPs and progressors exhibit comparable levels of T cell activation or apoptosis, comparable frequencies of Tregs, and equivalent proportions of CD16^+^ intermediate monocytes, which are linked to immune activation and inflammation.^147^^,^^149^^,^^150^^,^^152^^,^^153^ Conversely, other studies have documented lower expression of ISGs and lower levels of immune activation in T cells, reduced bystander CD4^+^ T cell apoptosis, and signs of attenuated intestinal disruption in VNPs.^9^^,^^140^^,^^143^ It is noteworthy that the characteristics of the control populations used for comparison with VNPs vary widely across different studies, potentially contributing to the conflicting results and introducing an additional layer of complexity in the results interpretation. Future studies in this dynamic field should be carefully designed to identify signatures specific to VNPs.

## Comparing natural SIV hosts and VNPs: Learnt lessons and future directions

The different scenarios of HIV/SIV infection that we compare in this review have converged in a similar non-progressing infection profile. In the case of natural SIV hosts, thousands of years of natural selection and coevolution between the virus and the host have resulted in harmless coexistence, as high persistent viral loads favor transmission but infected animals remain free of disease. In the case of pediatric VNPs, the nature of the immature and tolerogenic immune system during early life favors uncontrolled viral replication without triggering overreactive immune responses that could contribute to disease progression. Finally, the case of exceptional adult VNPs is particularly puzzling, as high levels of viral replication do not trigger the devastating effects on the immune system that would be expected in standard individuals due to multifactorial mechanisms.

**Figure 3:**
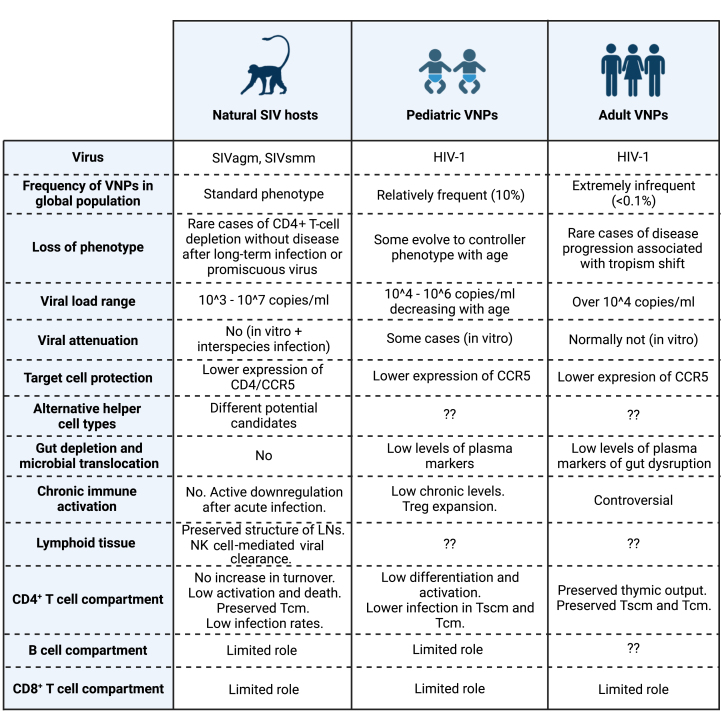
Comparison of the main features of viremic non-progression in HIV/SIV infection Natural SIV hosts, pediatric VNPs, and adult VNPs share a common infection profile as a result of different circumstances. In all cases, viruses normally preserve intact pathogenic potential, and the resistance to SIV/HIV pathogenesis is due to protective immune characteristics, some of them preserved across all conditions. However, the extremely low frequency of VNPs, especially among adult populations, and the scarcity of integrative studies on this phenotype have caused many questions to remain unanswered.

We have shown that the resistance to pathogenesis in the different settings that we analyze is generally not due to functional defects of viruses but due to protective characteristics of the host immune system. Importantly, some of these traits have been observed across non-progressing SIV and HIV infections and seem to be important for the development of a VNP-like phenotype, such as partial protection from infection of target cell populations. However, important knowledge gaps still remain for pediatric VNPs and the extremely infrequent adult VNPs, and we should not assume that the same exact immunological mechanisms observed in natural SIV hosts can be directly extrapolated to the other conditions (Figure 3). For example, a low degree of chronic immune activation and inflammation is a hallmark in studies on AGMs and SMs, and similar observations have been made on pediatric VNPs, while previous studies on adult VNPs showed contradictory results and have not clearly concluded whether low immune activation is also required for this phenotype, probably due to the small sample size and heterogeneity of the populations being assessed. Moreover, there is a notable absence of studies that assess the characteristics of tissue compartments crucial in HIV infection, such as gut and lymph nodes, whose preservation plays an essential role in natural SIV hosts. New studies involving larger cohorts of VNPs covering geographic, sexual, and viral subtype diversity, and encompassing a broader research scope are recommended to achieve a comprehensive understanding of the immune characteristics of these individuals. However, future research on the VNP phenotype and its NHP counterparts will have to adapt to conservational restrictions on natural SIV host research and the difficult identification of new cases of VNPs in the era of early ART initiation. Future studies on VNPs should therefore consider the combination of different approaches: (1) utilization of biobanks to retrospectively identify VNPs through inspection of clinical records and use of cryopreserved samples; (2) identification of new VNPs in settings where ART is not yet widely implemented; (3) prospective collection of samples from VNPs, with a special interest on tissue specimens; and (4) application of novel multiomics and machine learning technologies to maximize the information obtained from these scarce samples and unravel distinctive mechanisms driving resistance to HIV pathogenesis in this population.

Research on natural SIV hosts and VNPs has established a robust foundational framework for guiding the development of therapies and holds great promise as a valuable source of knowledge in the future. Strategies aimed at recovering CD4^+^ T cell counts in ART-suppressed immune non-responders should be preferentially directed toward subpopulations with high proliferative potential such as Tscm and Tcm.^154^ Specifically, the recovery of the Th17 compartment, which is severely and permanently damaged during HIV-1 infection, might benefit from therapies including IL-21 treatment, manipulation of IDO1-mediated Trp metabolism, or microbiome-targeted interventions. Also, induction of helper-like phenotypes in cells other than CD4^+^ T cells could also be explored as a complementary strategy. New therapeutic approaches aimed at modulating NK cell responses and promoting their internalization into lymph node follicles to clear infected cells, as observed in natural SIV hosts, offer promising potential to purge viral reservoirs in tissues. In this regard, IL-21-based therapies, which have been linked to the generation of protective NK cell phenotypes, hold great promise.^110^ Besides, strategies for reduction of chronic immune activation and inflammation, which still constitute a major burden for ART-treated people, should seek the neutralization of microbial translocation products such as LPS, downregulation of innate immune sensing pathways like TLR4 signaling, and downregulation of chronic IFN responses. Importantly, interventions targeting IFN response to reduce chronic immune activation might consider the simultaneous targeting of type-I IFNs and IFNγ, which has also been shown to contribute to maintain the upregulation of ISGs during chronic SIV infection.^155^

## Conclusions

The study of extreme phenotypes of HIV-1 infection that avoid the natural pathogenic progression has been historically a source of inspiration for the development of new therapeutic strategies based on their protective biological characteristics. The extraordinary case of adult and pediatric VNPs, who maintain normal CD4^+^ T cell counts despite elevated and persistent viral replication, together with their primate counterparts, the natural SIV hosts, is an excellent example with potential beneficial applicability to ART-treated PWH suffering from incomplete CD4^+^ T cell recovery or chronic immune activation. On the one hand, unraveling the mechanisms that allow VNPs to maintain the CD4^+^ T cell homeostasis could benefit immune non-responders, who represent 10%–40% of all ART-treated people,^1^ do not recover normal CD4^+^ T cell counts despite suppressed viremia, and suffer an increased risk of morbidity and mortality from AIDS and non-AIDS events. On the other hand, understanding how VNPs can mitigate the effects of chronic immune activation could contribute to alleviate the burden of chronic inflammation and its associated comorbidities, which constitutes a very active field of research, among PWH.

In this review, we have shown that lack of disease progression in viremic SIV infection is accomplished through a complex multifactorial mechanism that involves exceptional immunological features, some of which have been confirmed in parallel studies on VNP populations. However, important knowledge gaps on the biological underpinnings of VNPs still persist, and new studies that take advantage of recent multiomic technologies might contribute to complete our understanding of this infrequent but extremely valuable phenotype. The emulation of such protective characteristics holds great therapeutic potential to solve unmet needs in target populations affected by HIV-1.

## Acknowledgments

A.B.-G. was awarded the grant FPU17/04766 from the Spanish Ministry of Science, Innovation and Universities. Research in the J.M.-P. lab is supported by the 10.13039/501100004837Spanish Ministry of Science, Innovation and Universities (grants PID2022-139271OB-I00 and CB21/13/00063, Spain), NIH/10.13039/100000060NIAID (1UM1AI164561-01 and 1P01AI178376-01, United States), 10.13039/100015439Research Centres of Catalonia (10.13039/100015439CERCA, grant 2017 SGR 252, Spain), and Generalitat Valenciana (grant PROMETEO/2021/036, Spain). BioRender software was used for illustration.

## Author contributions

A.B.-G. designed the content, wrote the original draft, and edited the manuscript. M.C.P. and J.M.-P. revised and edited the manuscript. All authors contributed to the article and approved the submitted version.

## Declaration of interests

The authors declare no competing interests.
